# In Situ Dose Measurements in Brachytherapy Using Scintillation Detectors Based on the Al_2_O_3_:C, Al_2_O_3_:C,Mg, and GAGG:Ce Crystals

**DOI:** 10.3390/ma19010045

**Published:** 2025-12-22

**Authors:** Sandra Witkiewicz-Lukaszek, Janusz Winiecki, Bogna Sobiech, Mark Akselrod, Yuriy Zorenko

**Affiliations:** 1 Faculty of Physics, Kazimierz Wielki University in Bydgoszcz, Powstańców Wielkopolskich Street 2, 85-090 Bydgoszcz, Poland; 2 Franciszek Łukaszyk Oncology Center, Medical Physics Department, dr Izabeli Romanowskiej Street 2, 85-796 Bydgoszcz, Poland; sobiechb@co.bydgoszcz.pl; 3 Department of Oncology and Brachytherapy, Collegium Medicum in Bydgoszcz Nicholas Copernicus University in Toruń, Jagiellońska Street 13/15, 85-067 Bydgoszcz, Poland; 4 Department of Physics, Oklahoma State University, 145 Physical Sciences Building, Stillwater, OK 74078, USA; akselrod.mark@gmail.com

**Keywords:** fiber-optic detector, scintillation crystals, sapphire, garnets, brachytherapy, γ-rays

## Abstract

Currently, the use of scintillation crystals connected via optical fiber to a luminescence spectrometer (so-called fiber-optic dosimeters) offers a promising approach for real-time dosimetric measurements during brachytherapy treatments with γ-ray sources. This study aims to evaluate the applicability of fiber-optic dosimeters for in situ dose measurements during brachytherapy procedures, using Al_2_O_3_:C and Al_2_O_3_:C,Mg crystals, which have near-tissue density and effective atomic number (ρ = 3.99 g/cm^3^, Z_eff_ = 10.8), as well as heavy GAGG:Ce scintillation crystals (ρ = 6.63 g/cm^3^, Z_eff_ = 54.4). Radiation dose delivery was assessed through measurements of the resulting radioluminescence of the aforementioned scintillation crystals, connected via long optical fibers and recorded with highly sensitive, compact luminescence spectrometers. Measurements were performed in a dedicated phantom under clinical conditions at the Oncology Center in Bydgoszcz, Poland. The dosimeters were evaluated for in situ dose monitoring within the 0.5–8 Gy range during brachytherapy procedures using a ^192^Ir (392 keV) source. The results showed a clear linear relationship between the delivered radiation dose and the scintillation output measured by the fiber-optic detector. The Gd_3_Al_2.5_Ga_2.5_O_12_:Ce crystal detector exhibited excellent linearity, while the Al_2_O_3_:C and Al_2_O_3_:C,Mg crystal detectors also showed a nearly linear dose–response relationship.

## 1. Introduction

Radiotherapy achieves its therapeutic objective by delivering the prescribed ionizing radiation dose with high spatial accuracy to the tumor volume, while minimizing exposure of surrounding healthy tissues, particularly organs at risk (OARs). To meet this objective, robust quality assurance (QA) procedures must be applied to confirm that the treatment planning system (TPS) and treatment delivery system function accurately and remain within specified limits.

Patient-specific quality assurance (PS-QA) extends this verification process to the clinical environment, accounting for variables such as organ motion, anatomical changes, and applicator positioning uncertainties. These measurements can be performed prior to treatment or in situ, provided they do not perturb the intended dose deposition [1,2]. Robust PS-QA procedures are particularly critical in brachytherapy, where steep dose gradients near the source and the proximity of OARs leave little room for deviation [2].

In external beam radiotherapy (EBR), where targeted intensity modulation techniques currently dominate, 2D or 3D detector arrays are widely used for treatment verification. Such systems, employing either liquid ionization chambers or semiconductor elements, allow high-resolution spatial dose mapping and have become standard for QA in Intensity-Modulated Radiation Therapy (IMRT) and Volumetric Modulated Arc Therapy (VMAT) [1,2]. However, their application is limited to pre-treatment verification in air or phantoms, as they cannot be used in direct contact with the patient.

In contrast, brachytherapy, where radioactive sources are introduced into body cavities (intracavitary) or interstitially implanted, poses a distinct challenge. The extremely localized dose distribution renders point or planar detector arrays impractical, as 3D mapping within the patient’s body is unfeasible. Furthermore, only the escaping fraction of the emitted radiation can be detected externally, which restricts the accuracy of real-time in vivo dosimetry [2,3,4,5,6,7,8,9].

To address this limitation, point dose measurements may be performed by inserting miniature detectors into the body cavity, in close proximity to the radiation source and target volume. However, traditional detectors such as ionization chambers or semiconductor diodes are unsuitable due to the potential risk of electric shock and size constraints. Passive dosimeters such as thermoluminescent (TL) or optically stimulated luminescence (OSL) detectors, while safe and well-established, provide only retrospective dose information, precluding immediate correction of any detected irregularities [10,11,12].

Recently, significant research efforts have been directed toward developing *fiber-optic dosimetry* systems capable of real-time, in vivo dose monitoring in brachytherapy [3,4,5,6,13,14,15,16,17]. Such systems exploit the light-emitting properties of scintillating materials coupled to optical fibers, offering electrical safety, high spatial resolution, and minimal perturbation to the dose field. In early clinical trials, plastic scintillator-based *fiber optic detectors* (FODs) demonstrated deviations within ±10% from the planned dose in patient measurements [7], validating their potential for clinical implementation. Nevertheless, measurement discrepancies of up to 9% were attributed partly to detector displacement during irradiation, reflecting the importance of stable sensor positioning and robust calibration under steep dose gradients [7,16].

Subsequent studies have refined the FOD concept by optimizing scintillator composition and geometry to improve sensitivity, reduce stem effects (Cherenkov and fiber luminescence), and ensure compatibility with treatment imaging systems [3,4,5,6,18,19]. The choice of scintillator material dictates the detector’s optimal configuration: low-density, water-equivalent materials are suited for “*in front of the tumor*” placements, whereas high-density, high-Z_eff_ scintillators offer enhanced backscatter response in “*behind the tumor*” geometries [17,20,21].

In our previous works [19,20], fiber-optic detectors incorporating Ce^3+^-doped garnet scintillators such as Y_3_Al_5_O_12_ (YAG:Ce), Lu_3_Al_5_O_12_ (LuAG:Ce), and Gd_3_Al_2.5_Ga_2.5_O_12_ (GAGG:Ce) were investigated for high-dose-rate (HDR) brachytherapy applications. These materials exhibited excellent linearity, high scintillation efficiency, and emission spectra well matched to silicon-based photodetectors, allowing efficient light collection without ultraviolet optics. Furthermore, their superior radiation hardness, maintained under ion fluences exceeding 10^10^ ions/cm^2^ and γ-ray doses beyond 10^5^ Gy, supports their use in long-term clinical QA [19,20].

Parallel developments have highlighted the dosimetric versatility of aluminum oxide–based materials such as Al_2_O_3_:C and Al_2_O_3_:C,Mg, which have been extensively used in passive thermoluminescence (TL) and opticall-stimilated luminescence (OSL) dosimetry [10,12,21]. Their near-tissue equivalence (ρ = 3.99 g/cm^3^, Z_eff_ = 10.8), high sensitivity, and negligible signal fading make them attractive candidates for hybrid active–passive dosimetry systems. Recent implementations of μ-Al_2_O_3_:C,Mg radioluminescent films for two-dimensional, real-time QA in radiotherapy [12] further demonstrate the feasibility of integrating these materials into advanced optical dosimetry platforms.

Recent progress in the field has also addressed key limitations such as the “stem effect” in optical fibers [3,4,5,18], the need for multi-point dose mapping [9,17], and the adaptation of scintillation detectors for ultra-high dose rate (UHDR) or “FLASH” radiotherapy [13,18]. Novel stem-effect-free materials such as (Y,Yb)AG-based scintillators [18] and multi-probe FOD configurations [17] have shown promising results, offering higher accuracy and reduced signal contamination.

Building on these advances, the present study reports the latest achievements in developing an effective fiber-optic dosimetry (FOD) system that combines the advantages of established dosimetric and scintillation materials with radiation-hardened optical fibers and compact, high-sensitivity luminescent spectrometers for γ-ray dose detection in brachytherapy. The approach aims to establish a robust foundation for real-time, patient-specific QA, ultimately enhancing the safety and precision of modern brachytherapy treatments.

Although FOD systems used for in situ *dosimetry* are well known in the literature ([1,2,3,4,5,6,7,8,9,10,11,12,13,14,15,16,17,18,19,20,21] and Table 1), the discussion of advantages and disadvantages of specific detector systems mainly concerns the *choice of materials* (primarily plastic scintillators [2,3,4,5,6,7,8,9], LiF [12] and others [15,16]); the *construction of detectors* (e.g., selection of PMTs and optical fiber types); or the *readout methods* (OSL decay kinetics [10,11,12,16] or direct RL output [13,14,15,16,17,18] under additional excitation). However, the major advantages of the method proposed by us ([19,20]) are the *use of high-performance scintillation materials* with extremely high light yield (LY) [22,23,24], as well as the registration of *RL spectra* (rather than only total RL output) (Table 1). Such a dual approach enables an excellent *RL response* versus *delivered dose* and completely eliminates the influence of undesirable factors such as *stem effects*. It also allows the application of composite scintillators emitting simultaneously in different spectral ranges [22]. This is extremely important not only for X-ray or γ-rays single-source emitters such as ^192^Ir, but also for dose monitoring in mixed radiation fields (particles and quanta) through the simultaneous detection of spectra from different components of composite scintillators used for instant in *BNCT treatment procedures* [22]. For these reasons, the development of FOD systems based on our proposed approach opens the way for much broader application of efficient scintillation materials, including those suitable for the production of composite scintillators using the LPE method [22] or more advanced thin-film technologies.

## 2. Samples and Equipment

The compact FOD sensor heads, incorporating scintillation crystals sized between 1 × 1 × 0.5 mm and 2 × 2 × 1 mm, are well suited for brachytherapy applications. Their minimal dimensions enable straightforward integration into standard brachytherapy tools, such as urinary catheters, for real-time dose monitoring. The measured dose can then be compared with the planned dose distribution to confirm that the patient receives the prescribed treatment.

Due to the mentioned small-size crystal detectors, it is possible to incorporate them into the sensing treatment range by the different ways, e.g., coating the fiber, coupling it to the time, or embedding it within the fiber. Distinct advantages of such system include real-time dosimetry, small size and good spatial resolution.

Our approach, which utilizes a long optical fiber with a small-sized, well-known dosimetric or scintillation material attached as the FOD head, offers several advantages over previously developed materials and systems [2,3,4,5,6,7,8,9,10,11,12,13,14,15,16]. The first materials selected for investigation in this work were pieces of commercially available, well-known dosimetric crystals of carbon-doped and carbon–magnesium co-doped Al_2_O_3_ (sapphire) [10,21], grown by the Czochralski (Cz) method in the Stillwater Crystal Growth Division of Landauer Inc., Glenwood, IL, USA, under a highly reducing atmosphere in the presence of graphite. The second material was a piece of a commercially available, highly efficient scintillation crystal of GAGG:Ce garnet, produced by the Czochralski method at 1850 °C in an Ar + 1.5% O_2_ atmosphere by C&A Ltd., Sendai, Japan. The concentration of dopants in crystals were 0.1 at.% (C) and 0.2 at.% (Mg) [21] in sapphire and 0.21 at.% (Ce) in garnet [19,20].

To evaluate the luminescent behavior of the investigated materials, cathodoluminescence (CL) spectra were recorded at room temperature (RT) using a JEOL JSM-820 scanning electron microscope, Milano, Italy, operated at an electron beam energy of 20 kV. The emitted light was analyzed with a StellarNet grating spectrometer equipped with a CCD detector, StellarNet, Inc., Tampa, FL, USA, covering the 200–1200 nm range.

**Figure 1:**
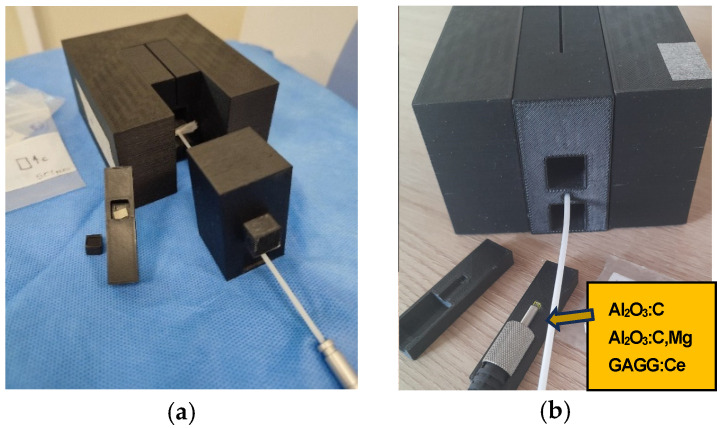
Phantom prepared from several HIPS bricks using a Zortrax M200 Plus printer, equipped with brachytherapy needles for positioning ^192^Ir γ-ray sources (**a**) and measuring system with scintillation crystal location (**b**).

All dose measurements with ^192^Ir γ-radiation were carried out using a High-Impact Polystyrene (HIPS) phantom fabricated with a Zortrax M200 Plus 3D printer, Zortax joint-stock company, Olsztyn, Poland (Figure 1a). The phantom was constructed to hold the FOD scintillation heads in contact with a 4.5 m optical fiber and the γ-ray source. Standard plastic brachytherapy needles, commonly used at the Oncology Center for positioning γ-ray sources, were also placed within the phantom (Figure 1b).

**Figure 2:**
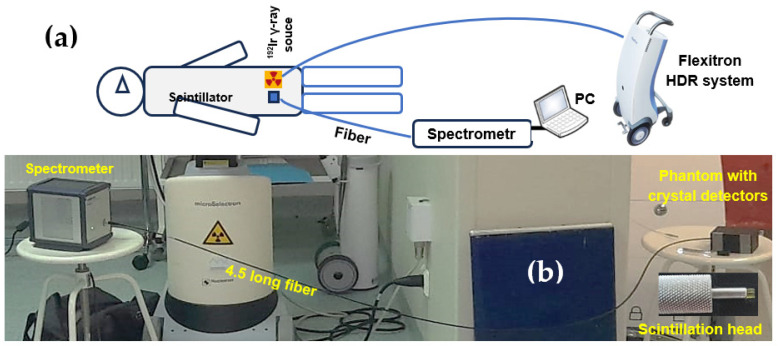
(**a**) Diagram illustrating the configuration used for in situ dose measurements within the patient’s body; (**b**) photograph of the measurement workstation in the brachytherapy treatment room at the Oncology Center in Bydgoszcz, showing the Al_2_O_3_:C scintillation head, a 4.5 m optical fiber, the Avantes AvaSpec HERO spectrometer, and a PC with the dedicated Avantes control software-AvaSoft Software (see also [17]).

The in situ dose-measurement system developed at the Oncology Center in Bydgoszcz is shown in Figure 2. A Flexitron HDR, Elekta, Stockholm, Sweden, unit with a ^192^Ir (392 keV) isotope was employed, with source activities of 5.85 Ci for Al_2_O_3_:C and Al_2_O_3_:C,Mg crystals and 7.15 Ci for GAGG:Ce. The device provides 40 channels and achieves 0.5 mm positioning accuracy. A high-sensitivity luminescence spectrometer, controlled from a separate operator room, was connected to the phantom via optical fibers: 4.5 m in length for Al_2_O_3_-based scintillators and 7 m for the GAGG:Ce detector. Each fiber terminated in a scintillator head positioned 1 cm from the ^192^Ir source (Figure 2b). The exposure times used to deliver doses of 0.5, 1, 2, 4, 6, and 8 Gy were 12, 23, 47, 96, 144, and 192 s for the Al_2_O_3_-based crystals, and 10, 23, 42, 82 122, and 161 s for the GAGG:Ce scintillator.

The radioluminescence (RL) spectra generated by the crystal detectors were recorded using an AvaSpec-HERO spectrometer (Avantes, Nynomic AG), Avantes B.V., Apeldoorn, The Netherlands. For the sapphire-based crystals, the spectral range was 320–600 nm, whereas for the garnet crystal it extended from 320–800 nm. Spectra were acquired with a 700 ms integration time throughout the entire irradiation period corresponding to doses between 0.5 and 8 Gy. The absence of signal below 320 nm results from the UV cutoff of the spectrometer’s glass optics.

## 3. Dose Distribution Prediction and Verification

**Figure 3:**
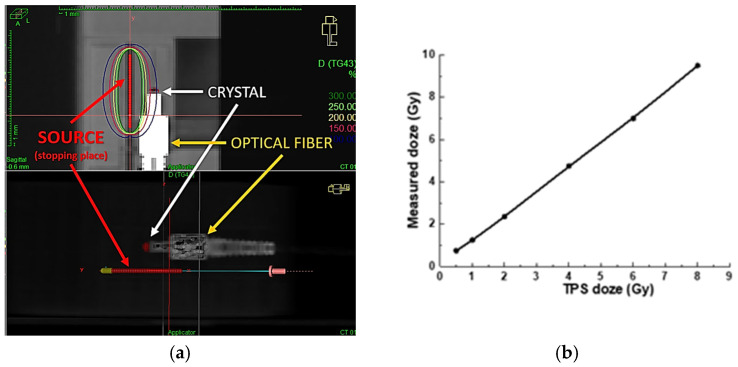
(**a**) Visualization of the planned dose distribution in the phantom generated by Oncentra Brachy TPS. The top panel shows a sagittal CT slice, and the bottom panel presents a 3D rendering. The Al_2_O_3_:Ce detector was located at a 1 cm distance from the radiation source. (**b**)—Dose comparison between measurements from a Farmer ionization chamber and values predicted by the TPS. Reprinted from Ref. [20].

The Treatment Planning System (TPS) used in our facility is ONCENTRA 4.6.3 from Elekta company, Stockholm, Sweden [23]. It enables calculation of the dose distribution received from point sources of radiation, i.e., ^192^Ir isotope, based on current tomographic images of the patient. Computed tomography (CT) scans are performed after inserting the applicators into the body cavities (or interstitial needles). For the purposes of the experiment, a scintillation detector should also be inserted along with the applicators, which can also be seen in the scans in Figure 3a.

In both the phantom experiment and the treatment planning procedures involving real patients, it is assumed that the distance between the detector and the radiation source remains constant throughout the entire treatment. However, this assumption may not always hold true during actual radiation delivery, potentially leading to discrepancies in the measured dose. To achieve high measurement precision, the detector must demonstrate satisfactory repeatability under identical conditions. Both the detector’s accuracy and the precision of its positioning are crucial for assessing the overall performance of the proposed methodology.

Figure 3b presents the results obtained during a phantom experiment using a conventional PTW 30010 Farmer, PTW Freiburg GmbH, Freiburg, Germany, ionization chamber for dose measurement. The expected dose values, calculated using the ONCENTRA TPS 4.6.3, ranged from 0.5 to 8 Gy. As shown in Figure 3b, our experimental setup enables linear dose delivery within this range, consistent with the TPS predictions, as verified by the readings from the Farmer-type chamber. These results confirm that, in the next phase, we can confidently replace the ionization chamber with our fiber-optic detector, ensuring accurate and reliable radiation dose delivery. Meanwhile, due to the significant difference in dimensions between the Farmer chamber and our scintillation crystal, we used custom-designed (3D-printed) adapters to ensure identical positioning during measurements (see Figure 1).

## 4. Experimental Results

**Figure 4:**
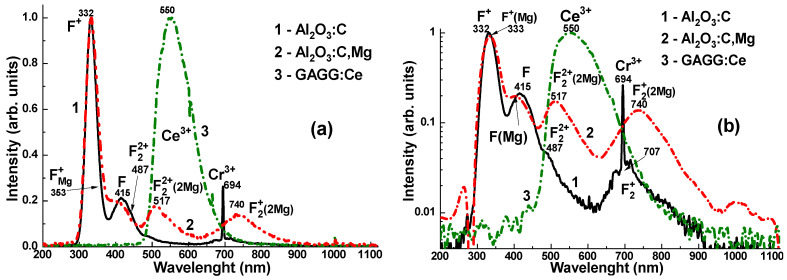
Normalized CL spectra of Al_2_O_3_:C (1), Al_2_O_3_:C,Mg (2) and GAGG:Ce (3) crystal detectors in linear (**a**) and logarithmic (**b**) scales.

The CL spectra of the Al_2_O_3_:C, Al_2_O_3_:C,Mg, and GAGG:Ce crystal detectors are presented in Figure 4. The emission spectra of the Al_2_O_3_:C crystal (Figure 4a,b, curves 1) represent the superposition of the dominant F^+^ and F centers’ luminescence bands, peaked at 332 and 415 nm, respectively, and low-intensity bands peaked at 487 and 707 nm, corresponding to the luminescence of F_2_^2+^ and F_2_^+^ pair centers [10,21]. Adding Mg^2+^ dopant to the sapphire host causes a notable modification of the electronic structure of F-like emission centers in the Al_2_O_3_:C,Mg crystal [21]. Namely, the emission spectra of this crystal (Figure 4a,b, curves 2) demonstrate the superposition of the dominant luminescence of F_Mg_^+^ centers (F^+^ at Mg dopant) in the band peaked at 333 nm, the emission of F_Mg_ centers (F at Mg dopant) in the band peaked around 415 nm, as well as quite intensive luminescence bands in the visible range, peaked at 517 nm and 740 nm, corresponding to F_2_^2+^(2Mg) and F_2_^+^(2Mg) aggregate centers, respectively [21].

However, it is worth noting that the spectral positions of the F_Mg_^+^ and F_Mg_ emission bands in the 400–450 nm range are well overlapped with the absorption/excitation band of the F_2_^2+^(2Mg) centers, peaked at 435 nm [21]. On the other hand, the green emission band of the F_2_^2+^(2Mg) aggregate centers at 517 nm partly overlaps with the absorption/excitation band of the other F_2_^+^(2Mg) aggregate centers at 620 nm [21]. For this reason, the luminescence of the F_Mg_^+^ and F_Mg_ centers also excites the emission of the F_2_^2+^(2Mg) and F_2_^+^(2Mg) centers in the Al_2_O_3_:C,Mg crystal [21]. In addition, it is known that the F^+^, F, F_2_^2+^, and F_2_^+^ centers in the Al_2_O_3_:C crystal, as well as the F_Mg_^+^, F_Mg_, F_2_^2+^(2Mg), and F_2_^+^(2Mg) centers in the Al_2_O_3_:C,Mg crystal, act as very efficient trapping centers [10,21]. As a result, energy transfer from the sapphire host to oxygen defect–related emission centers in the UV and visible ranges may be delayed when using Al_2_O_3_:C or Al_2_O_3_:C,Mg crystals as scintillators.

**Figure 5:**
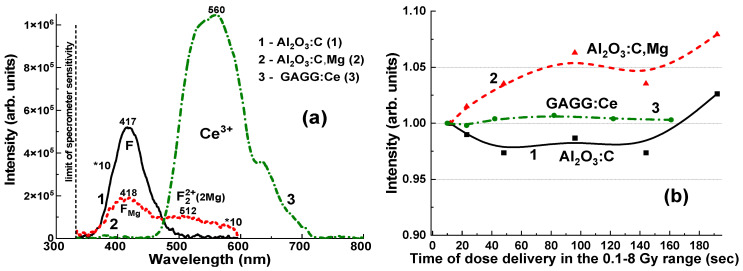
(**a**)—Integrated RL spectra of Al_2_O_3_:C (1) and Al_2_O_3_:C,Mg (2) crystal detectors in the 320–600 nm range, and the GAGG:Ce (3) crystal detector in the 320–800 nm range, recorded during the delivery of an 8 Gy dose from a ^192^Ir (392 keV) γ-ray source. (**b**)—Dependence of the stability of the F (1) and FA (2) emission bands in Al_2_O_3_:C and Al_2_O_3_:C,Mg crystals, respectively, and the Ce^3+^ emission band (3) in GAGG:Ce crystal, on the irradiation time for dose delivery ranging from 0.5 to 8 Gy.

**Figure 6:**
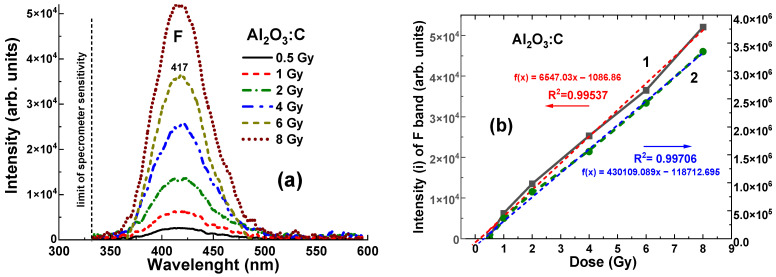
(**a**)—Dependence of the intensity of RL spectra for the Al_2_O_3_:C crystal detector on the radiation dose in the 0.5–8 Gy range. (**b**)—Relationship between the RL band intensity of F centers (1) and the integral area of the RL spectra (2) as a function of the irradiation dose. The dashed lines represent the best linear-fit approximations of these dependences *f*(*x*) = 6527*x* − 0.065 and *f*(*x*) = 430,109*x* − 0.125, respectively, with the R^2^ values indicating the goodness of fit.

**Figure 7:**
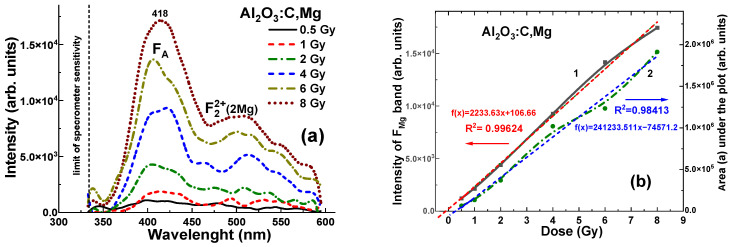
(**a**)—Dependence of the intensity of RL spectra for the Al_2_O_3_:C,Mg crystal detectors on the radiation dose in the 0.5–8 Gy range. (**b**)—Relationship between the RL peak intensity of F_A_ centers (1) and the integral area of the RL spectra (2) as a function of irradiation dose. The dashed lines represent the best linear-fit approximations *f*(*x*) = 2241 × *x* − 0.04 and *f*(*x*) = 22,703 × *x* − 0.227, respectively, and the R^2^ values indicate the goodness of fits.

Interactions between various defect-related centers may also contribute to changes in the RL intensity of the F-like emission bands, depending on the measurement duration (Figure 5b). Specifically, the Al_2_O_3_:C crystal detector exhibits only a slight variation in the F-center emission band intensity, ranging from 97.3% to 102.6%, as the irradiation time increases from 12 s to 192 s and the delivered dose rises from 0.5 to 8 Gy (Figure 5b, curve 1). In contrast, the F_Mg_ and F_2_^2+^(2Mg) emission bands show a more pronounced increase, from 100% to 108%, under the same irradiation conditions (Figure 5b, curve 2). These effects can also significantly influence the measurement of the total emission intensity for Al_2_O_3_:C and Al_2_O_3_:C,Mg crystals, which remains approximately proportional to the exposure time for the applied dose (Figure 6 and Figure 7).

The emission spectrum of the GAGG:Ce crystal (Figure 5a, curve 3) exhibits a strong yellow-green luminescence, with a peak at 550 nm in the visible range, corresponding to the 5d_1_–4f transitions of Ce^3+^ ions in the garnet matrix [24,25,26]. Additionally, a weak band is observed at 385 nm, attributed to F^+^ centers [27,28]. Notably, the GAGG:Ce crystal shows minimal emission from antisite defect (AD)-related centers, specifically Gd_Ga_ and Gd_Al_, in the UV region [29]. This can be attributed to the band gap engineering [30] and the depopulation (or “burning”) of the AD centers’ energy levels via the lower conduction band states formed predominantly by the 3d levels of Ga^3+^ cations [31]. This behaviour may offer a distinct advantage of the GAGG:Ce crystal over its Al_2_O_3_-based counterparts, particularly in terms of the RL intensity stability with respect to irradiation time (dose) (Figure 5b, curves 1–3).

It is worth noting that even with relatively low density and $Z_{\mathrm{eff}}$ values, the Al_2_O_3_:C crystal detector shows a clearly measurable level of RL, recorded in the 320–600 nm range, at all γ-ray radiation doses from 0.5 to 8 Gy, which corresponds to the range of (2.6–5.2) × 10^4^ units (Figure 6a). The dependences of the RL intensity (I) of the F band and the area (A) between the RL spectra and the *x*-axis for the Al_2_O_3_:C crystal detector on the radiation dose within the mentioned range are shown in Figure 6b. The data were fitted with dashed linear lines and the R^2^ value in Figure 6b and Figure 7b indicates the goodness of fit of the model to the observed data. R^2^ values range from 0 to 1, where R^2^ = 0 indicates that the model explains none of the variability in the data, and R^2^ = 1 means the model perfectly captures the data’s variability. As shown in Figure 6b, the relationships between intensity (I) and dose, and between integral area (A) and dose for the Al_2_O_3_:C crystal detector exhibit strong linearity in the range of 1–8 Gy. These dependencies are described by the equations *f*(*x*) = 2241*x* − 0.04 and *f*(*x*) = 22,703*x* − 0.227, respectively, where *x* is the dose in Gy and *f*(*x*) represents the intensity or the integral area. Furthermore, slight deviations from ideal signal–dose linearity are observed. They are characterized by the intercept values of −0.065 Gy and −0.125 Gy, respectively, corresponding to *x* = 0 Gy (Figure 6b).

Contrary, the Al_2_O_3_:C,Mg crystal detector with same density and effective atomic number, shows significantly lower level of RL (to 3–5 time), registered in the 320–600 nm range. For all γ radiation doses in the 0.5–8 Gy range, the maximum RL intensity at 418 nm falls within the range of (0.12–1.7) × 10^5^ units (Figure 7a). The dependences of the RL intensity (I) of the F_Mg_ band and area (A) between RL spectra and *x* axis for Al_2_O_3_:C,Mg crystal detector on the radiation dose in the mentioned range is shown in Figure 7b. These dependencies are described by the equations *f*(*x*) = (2241 × *x* − 0.04) Gy and *f*(*x*) = (22,703 × *x* − 0.227) Gy, respectively, where *x* is the dose in Gy and *f*(*x*) represents the intensity I or the integral area A. Meanwhile, in slight contrast to the Al_2_O_3_:C crystals, the corresponding relationships for the Al_2_O_3_:C,Mg crystal detector show a slightly larger deviation from ideal signal–dose linearity (Figure 7b). These deviations can be characterized by the intercept values of +0.04 Gy and −0.227 Gy, respectively, corresponding to *x* = 0 Gy (Figure 7b).

**Figure 8:**
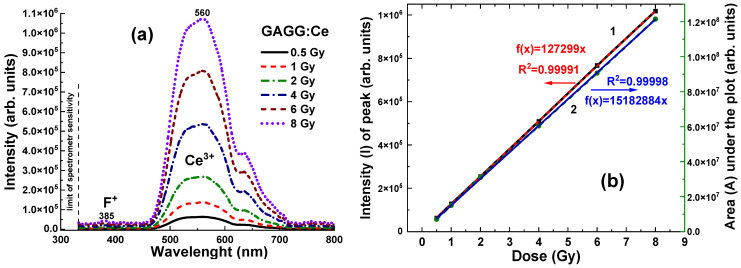
(**a**)—Dependence of the RL spectra intensity for the GAGG:Ce crystal detector on the radiation dose in the 0.5–8 Gy range (see also [17]). (**b**)—Relationship between the RL peak intensity of Ce^3+^ ions (1) and the RL band intensity (2) as a function of irradiation dose. The dashed lines represent the close-to-ideal linear-fit approximations, *f*(*x*) = 127,299 × *x* and *f*(*x*) = 15,182,884 × *x*, respectively, and the R^2^ values indicate the goodness of fit. Reprinted from Ref. [20].

Due to its high density and effective atomic number, the GAGG:Ce crystal detector exhibits a significantly higher RL intensity, recorded in the 320–800 nm range, across all γ radiation doses in the 0.5–8 Gy range, with values ranging from (0.65–1.1) × 10^6^ units (Figure 8a). The relationships between the RL intensity (I) of the Ce^3+^ band and the integral area (A) of the RL spectra, as a function of radiation dose, for the GAGG:Ce scintillator are shown in Figure 8b. In contrast to Al_2_O_3_:C and Al_2_O_3_:C,Mg crystals, the dose-dependent relationships for the GAGG:Ce crystal detector exhibit excellent linearity across the entire 0.5–8 Gy dose range (Figure 8b).

## 5. Discussion

An analysis of existing alternatives for accurate dosimeters in brachytherapy indicates that future advancements will likely center on in vivo dosimetry systems capable of real-time dose monitoring (Table 1). We believe that FOD, which combines a scintillator and optical fiber, will be instrumental in in vivo dosimetry for radiation therapy, enabling direct, real-time monitoring of the radiation delivered to both the tumor and surrounding critical structures.

This study aims to assess the feasibility of using fiber-optic dosimeters for in situ dose measurements during brachytherapy, specifically utilizing Al_2_O_3_:C and Al_2_O_3_:C,Mg crystals, which have near-tissue density and effective atomic numbers (ρ = 3.99 g/cm^3^, Z_eff_ = 10.8), alongside a heavier GAGG:Ce scintillation crystal (ρ = 6.63 g/cm^3^, Z_eff_ = 54.4).

During the experimental evaluation, significant variations were observed in both the intensity and the area of the luminescence bands across all tested crystals, depending on the radiation doses applied in the 0.5–8 Gy range. These changes in peak intensity and area are critical factors in assessing the sensitivity and accuracy of dose measurements. The variations observed in the “peak intensity/area versus dose” relationships highlight the distinct radiation response characteristics of each crystal. This suggests that the choice of Al_2_O_3_:C, Al_2_O_3_:C,Mg, or GAGG:Ce detectors can be optimized for specific brachytherapy applications. Specifically, these differences imply that each material may offer unique advantages, depending on the target dose range and the specific measurement requirements.

**Figure 9:**
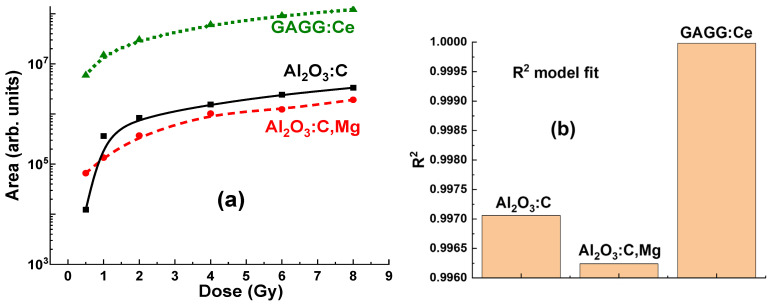
(**a**)—Comparison of the RL spectra area values, plotted on a logarithmic scale, as a function of irradiation dose (0.5–8 Gy) for Al_2_O_3_:C, Al_2_O_3_:C,Mg, and GAGG:Ce crystal detectors. (**b**)—Comparison of the R^2^ values for the model fits of the three detector types. An R^2^ value close to 1 indicates that the model accurately describes the variability in the data.

The significant difference in RL intensity observed among the crystals suggests that the Al_2_O_3_:C,Mg detector is notably less efficient in its RL response to γ-ray radiation (see Figure 5a, Figure 7a and Figure 9). In contrast, the Al_2_O_3_:C crystal detector with same Z_eff_ appears to enhance the RL efficiency (Figure 5a, Figure 6a and Figure 9), likely due to higher concentration of F centers in comparison with content F_Mg_ and F_2_^2+^(2Mg) centers, emitting in the 320–600 nm range. However, the Al_2_O_3_:C,Mg crystal detector shows a slightly more pronounced dose-intensity relationship compared to Al_2_O_3_:C detector on the 0.5–2 Gy range (Figure 7b and Figure 9), indicating that the luminescent output of first detector is more sensitive even to small changes in radiation dose. This characteristic makes Al_2_O_3_:C,Mg crystal a potentially more reliable material for applications where precise low-dose measurement is crucial. The more linear dose-dependency also suggests that Al_2_O_3_:C,Mg can be effectively used to detect small variations in radiation exposure, providing more accurate and detailed data in comparison with Al_2_O_3_:C crystal counterpart.

More generally, the observation of some nonlinearity in the dose–response relationship for the Al_2_O_3_:C and Al_2_O_3_:C, Mg detectors in 0.5–8 Gy range is an important finding (see Figure 6b and Figure 7b). This non-linearity may suggest that at lower or higher doses, the energy absorption and transfer mechanisms within the crystals are not directly proportional to the dose. Such behavior could be attributed to the specific energy transfer processes from the excited Al_2_O_3_ host to various defect-related emission centers in these crystals. Understanding and accounting for this non-linearity is essential when using Al_2_O_3_:C and Al_2_O_3_:C,Mg detectors in applications that require precise dose measurement, particularly when the detectors are placed before the target.

Compared to Al_2_O_3_:C and Al_2_O_3_:C,Mg detectors, the GAGG:Ce crystal detector (Figure 8 and Figure 9) presents a promising balance of physical and luminescent properties, making it well-suited for dosimetric applications. Specifically, the GAGG:Ce crystal demonstrates a more linear dose–response, particularly in the low-dose range where non-linearity can be an issue (Figure 8b and Figure 9a). The GAGG host, with its high density and effective atomic number, provides excellent photon interaction and energy absorption characteristics. These properties make GAGG:Ce an ideal material for applications requiring a compromise between tissue-equivalent materials and the high sensitivity (but lower efficiency) of Al_2_O_3_:C and Al_2_O_3_:C,Mg crystals for γ-ray detection.

The excellent linear response of the GAGG:Ce crystal detector to irradiation dose (Figure 8b and Figure 9a) enables more predictable dosimetric readings, without the pronounced non-linearity observed in Al_2_O_3_:C and Al_2_O_3_:C,Mg detectors at both low and high doses. This reduces the need for complex corrections in the low-dose range. As a result, the GAGG:Ce crystal is a reliable choice for applications requiring consistent performance across a wide dose range. Furthermore, the GAGG:Ce crystal detector offers a good balance of luminescent efficiency and enhanced linearity, making it an attractive option for practical dosimetry, especially in cases where the detector is positioned behind the target.

## 6. Conclusions

Our recent investigation shows that Al_2_O_3_:C, Al_2_O_3_:C,Mg, and GAGG:Ce single crystals possess excellent luminescent and scintillation characteristics, indicating their suitability for real-time dose monitoring in brachytherapy procedures.

Among the tested materials, the Al_2_O_3_:C crystal detector showed better scintillation performance than the Al_2_O_3_:C,Mg crystals, despite having the same density ρ = 3.99 g/cm^3^ and effective atomic number Z_eff_ = 10.8. Specifically, Al_2_O_3_:C crystals exhibited a more intense radioluminescence (RL) signal under a ^192^Ir from (392 MeV) γ-ray excitation in the 0.5–8 Gy range. This improved efficiency suggests that the Al_2_O_3_:C crystal detector is particularly well-suited for high-dose radiation environments, offering more accurate and responsive measurements compared to its Al_2_O_3_:C,Mg counterpart. On the other hand, Al_2_O_3_:C,Mg crystals demonstrated a more pronounced and reliable dose–RL intensity relationship in the 0.5–2 Gy range, making them especially advantageous for applications that require precise dose measurements at lower radiation levels.

We observed that the GAGG:Ce crystal detector shows strong potential for dosimetric use. Its relatively high density (ρ = 6.63 g/cm^3^) and effective atomic number (Z_eff_ = 54.4) provide sufficient photon interaction and energy absorption, delivering adequate sensitivity without requiring extensive corrections across both low and high dose ranges. Additionally, the GAGG:Ce detector exhibits a highly linear dose–response behavior, in contrast to Al_2_O_3_:C and Al_2_O_3_:C,Mg detectors, which can show deviations from linearity at extreme dose levels. These characteristics make GAGG:Ce a dependable and versatile option for applications that demand stable and accurate performance over a wide range of radiation doses.

In summary, all scintillation crystals under study are promising materials for in situ dose measurement in brachytherapy. The differences observed between these materials provide valuable information for selecting the most appropriate type of crystal based on specific clinical needs and radiation dose requirements. Namely, the scintillation crystals based on the doped Al_2_O_3_ sapphire with close-to tissue density can be used in any location between the radiation source and target because they do not interfere with dose distribution. However, in the cases of radiation therapy where the detector can be located behind the target, the use of heavy, high-density and high-Z_eff_ GAGG:Ce scintillators is strongly preferred.

## Figures and Tables

**Table 1:** Materials and Measurement Methods for Fiber-Optic–Based Dosimetry in Brachytherapy Using RL/OSL/TL Response. Not applicable—the method does not involve RL signal detection; scintillation-only measurement (no analisis of RL spectra/OSL decay kinetic/or TL glow curves).

First Author (Year)/Ref.	Detector Material(s)	Measurement Method	RL Signal Type/Notes
Woulfe (2016) [1]	Various fiber-optic dosimeters: plastic scintillators, doped crystals	Review of in vivo dosimetry techniques based on RL and OSL	Mainly integrated RL (review-level); no spectral RL used
Fonseca (2020) [2]	Scintillators, OSL fibers, RL materials	Review of in vivo brachytherapy dosimetry	General integrated RL; no spectral analysis
Veronese (2024) [3]	RL fibers: Al_2_O_3_:C, LaBr_3_, doped crystals	Review of RL-based fiber dosimetry	Integrated and spectral RL; spectral analysis enables material characterization
Soares (2006) [4]	Fiber-optic scintillator	Dose measurements using scintillation	Not applicable—scintillation only; no RL/OSL/TL analises
Kirov (1999) [5]	Plastic scintillators	2D scintillation dosimetry	Not applicable—scintillation only
Lambert (2006) [6]	Plastic scintillator	Real-time scintillation dosimetry in HDR	Not applicable—scintillation only
Suchowerska (2011) [7]	Plastic scintillators in urethral catheter	Clinical scintillation dosimetry	Not applicable—scintillation only
Therriault-Proulx (2011) [8]	Plastic scintillation detectors	Real-time dosimetry in ^192^Ir HDR	Not applicable—scintillation only
Linares Rosales (2020) [9]	Multipoint plastic scintillator	HDR ^192^Ir source tracking	Not applicable—scintillation only
Andersen (2009) [10]	Al_2_O_3_:C (RL + OSL)	Online RL and OSL in ^192^Ir HDR	Integrated RL; spectral RL not applied clinically
Kertzscher (2014) [11]	Point scintillation detector	Adaptive real-time dosimetry	Not applicable—scintillation only
Johansen (2018) [12]	Time-resolved fiber-optic detectors	Time-resolved scintillation dosimetry	Not applicable—scintillation only
Nascimento (2020) [13]	μ-Al_2_O_3_:C,Mg RL films	2D real-time RL imaging	Integrated RL for QA; no spectral RL applied
Jaselske (2017) [17]	LiF fiber scintillator	Catheter-based scintillation dosimetry	Not applicable—scintillation only
Belley (2018) [15]	Fiber-based scintillator	Real-time dose-rate monitoring	Not applicable—scintillation only
Vanreusel (2022) [18]	Point plastic scintillator	UHDR scintillation dosimetry	Not applicable—scintillation only
Cova (2025) [14]	(Y,Yb)AG scintillator crystals	UHDR dosimetry without stem effect	Not applicable—scintillation only
Gonod (2023) [16]	Six-probe scintillator	Multipoint HDR brachytherapy	Not applicable—scintillation only
Sobiech (2024) [19]	Doped crystal scintillators	In vivo dosimetry	Spectrally resolved RL, clinically validated
Witkiewicz-Łukaszek (2025) [20]	Ce^3+^-doped garnet crystals	Real-time scintillation dosimetry	Spectrally resolved integrated RL
Akselrod (2011) [21]	Al_2_O_3_:C, Al_2_O_3_:C,Mg (OSL/TD)	Overview of OSL/TD dosimetry	Spectral RL discussed; not applied clinically (mainly material characterization)

## Data Availability

The original contributions presented in this study are included in the article. Further inquiries can be directed to the corresponding authors.
